# Supplementary material for: Longitudinal evaluation of the biodistribution and cellular internalization of the bispecific CD3xTRP1 antibody in syngeneic mouse tumor models
Source: J Immunother Cancer. 2023 Oct 29;11(10):e007596. doi: 10.1136/jitc-2023-007596 (PMC10619024; doi:10.1136/jitc-2023-007596)
Supplement: Supplementary data [file jitc-2023-007596supp001.pdf]

SUPPLEMENTAL MATERIAL

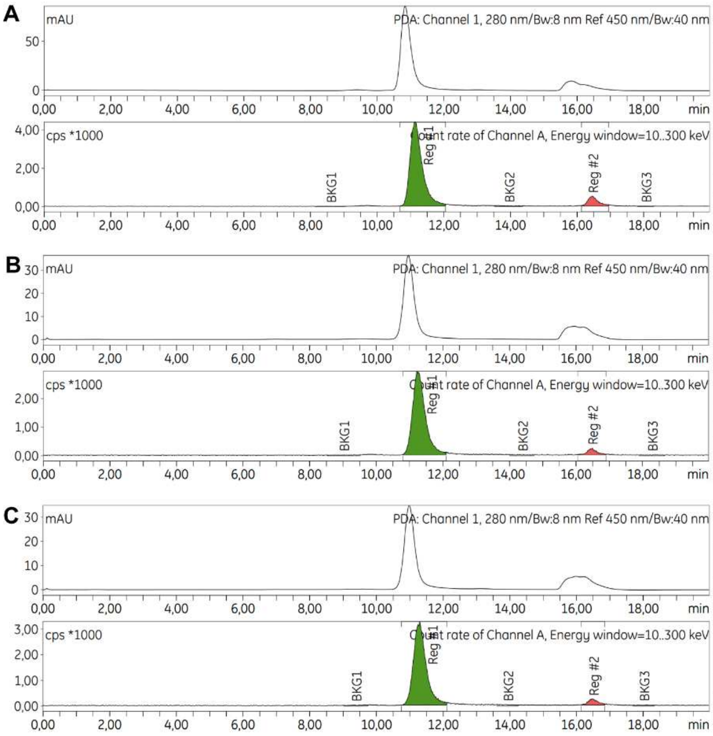

A

Substance	R/T min	Type	Area Counts	%Area %
Reg #1	11,13	DD(M)	112287,1	91,07
Reg #2	16,45	DD(M)	11014,3	8,93
Sum in ROI	-	-	123301,4	100,00
Area (total)	-	-	127788,9	-
BKG1 (CPS)	-	-	13,67	-
BKG2 (CPS)	-	-	35,92	-
3 BKG-ROIs	-	-	23,86	-
Remainder	-	-	4487,55	3,51

B

Substance	R/T min	Type	Area Counts	%Area %
Reg #1	11,25	DD(M)	77529,06	94,60
Reg #2	16,47	DD(M)	4426,69	5,40
Sum in ROI	-	-	81955,75	100,00
Area (total)	-	-	84485,14	-
BKG1 (CPS)	-	-	21,800	-
BKG2 (CPS)	-	-	31,512	-
3 BKG-ROIs	-	-	25,812	-
Remainder	-	-	2529,39	2,99

C

Substance	R/T min	Type	Area Counts	%Area %
Reg #1	11,28	DD(M)	84968,70	94,56
Reg #2	16,48	DD(M)	4888,16	5,44
Sum in ROI	-	-	89856,86	100,00
Area (total)	-	-	87933,67	-
BKG1 (CPS)	-	-	29,930	-
BKG2 (CPS)	-	-	47,026	-
3 BKG-ROIs	-	-	35,891	-
Remainder	-	-	-1923,19	-2,19

Figure S1. Purity of bsAbs assessed by HPLC. HPLC profiles of ^{111}In-labeled (at A_s: 1 MBq/µg) CD3xTRP1 **(A)**, CD3xMock **(B)**, and TRP1xMock **(C)** with 2.5 mM EDTA and supplemented with unlabeled bsAb to a final concentration of 0.5 mg/ml. [^{111}In]In-bsAb (Reg #1) and [^{111}In]In-EDTA (Reg #2) were separated using a SEC-3000 (Phenomenex) column with as a mobile phase of 0.1 M Na_3PO_4, 0.1 M Na2SO4, and 10% isopropanol at a flow rate of 0.7 ml/minute. For each bsAb, we show the protein detected by UV detector at 280 nm (upper panel), and measured radiosignal in counts per second (CPS, lower panel). The accompanying table shows the retention time (R/T), the activity count (Area Counts), and the % of total activity (%Area) in the integrated areas for [^{111}In]In-bsAb (Reg #1) and [^{111}In]In-EDTA (Reg #2).

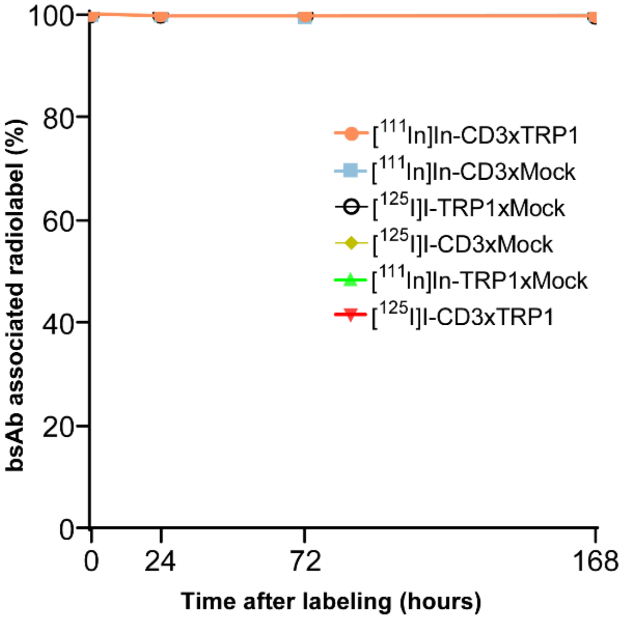

Figure S2. Stability of ^{111}In-bsAbs and ^{125}I-bsAbs over time. Stability of radionuclide:bsAb association of PD10 purified ^{111}In- and ^{125}I-labeled CD3xTRP1, CD3xMock, and TRP1xMock at 0, 24, 72, and 168 hours stored in PBS/0.5%BSA/0.1%Tween80 at 4°C, as assessed by iTLC.

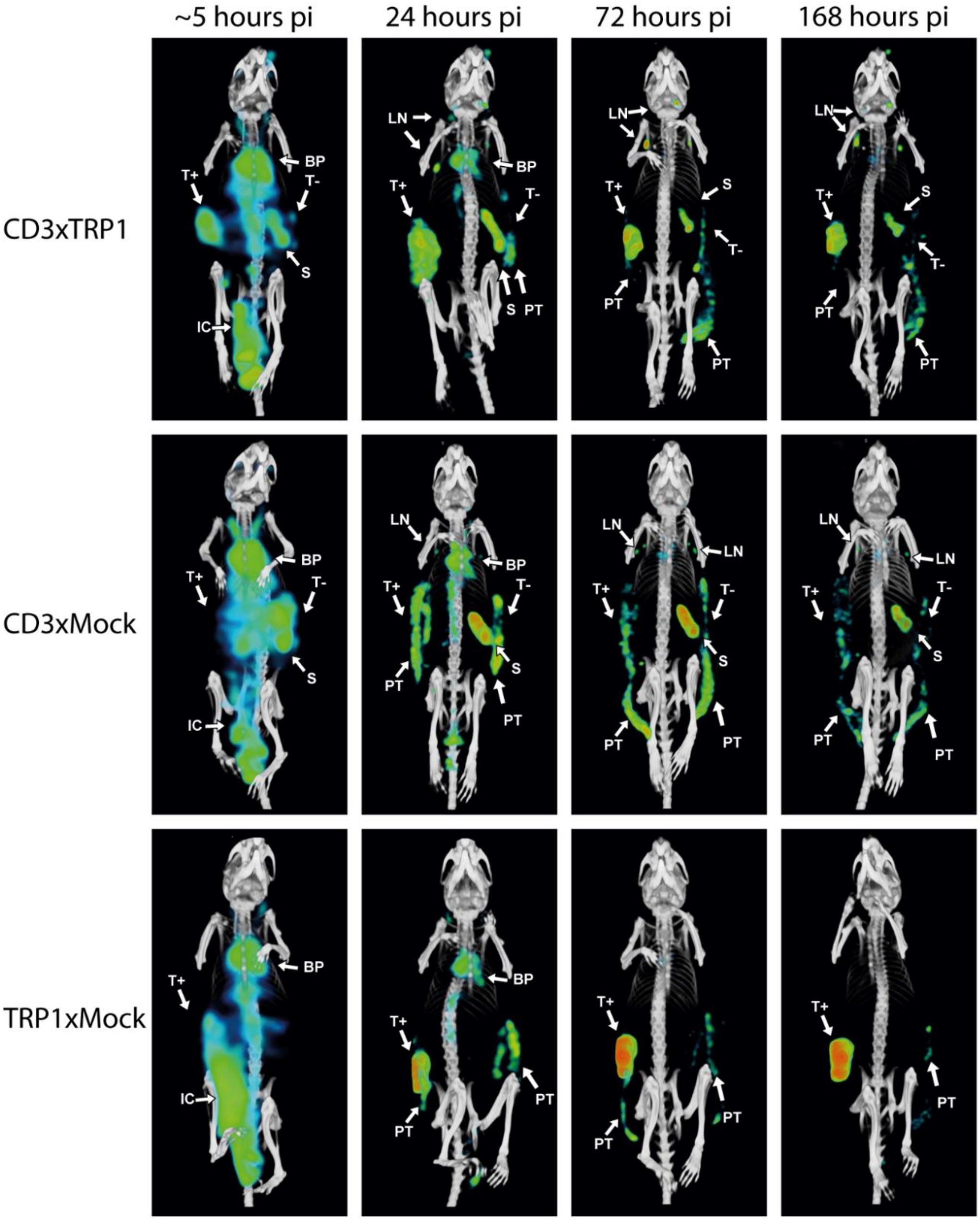

Figure S3. SPECT/CT images showing the *in vivo* biodistribution of CD3xTRP1, CD3xMock, and TRP1xMock over time. Representative MIPs of the *in vivo* biodistribution of 12.5 µg [^{111}In]In–CD3xTRP1, [^{111}In]In–CD3xMock, and [^{111}In]In–TRP1xMock in C57BL/6J mice bearing KPC3 and KPC3-TRP1 tumors on contralateral flanks at ~5, 24, 72, and 168 hours post intraperitoneal injection. Tissue uptake of [^{111}In]In–bsAbs is visualized with MIPs generated from microSPECT/CT data using the same thresholds at all time point. Indicated tissues with [^{111}In]In-bsAb uptake are: blood pool (BP), intraperitoneal cavity (IC), KPC3-TRP1 tumor (T+), KPC3 tumor (T-), spleen (S), lymph nodes (LN), and peri- and extratumoral accumulation (PT).

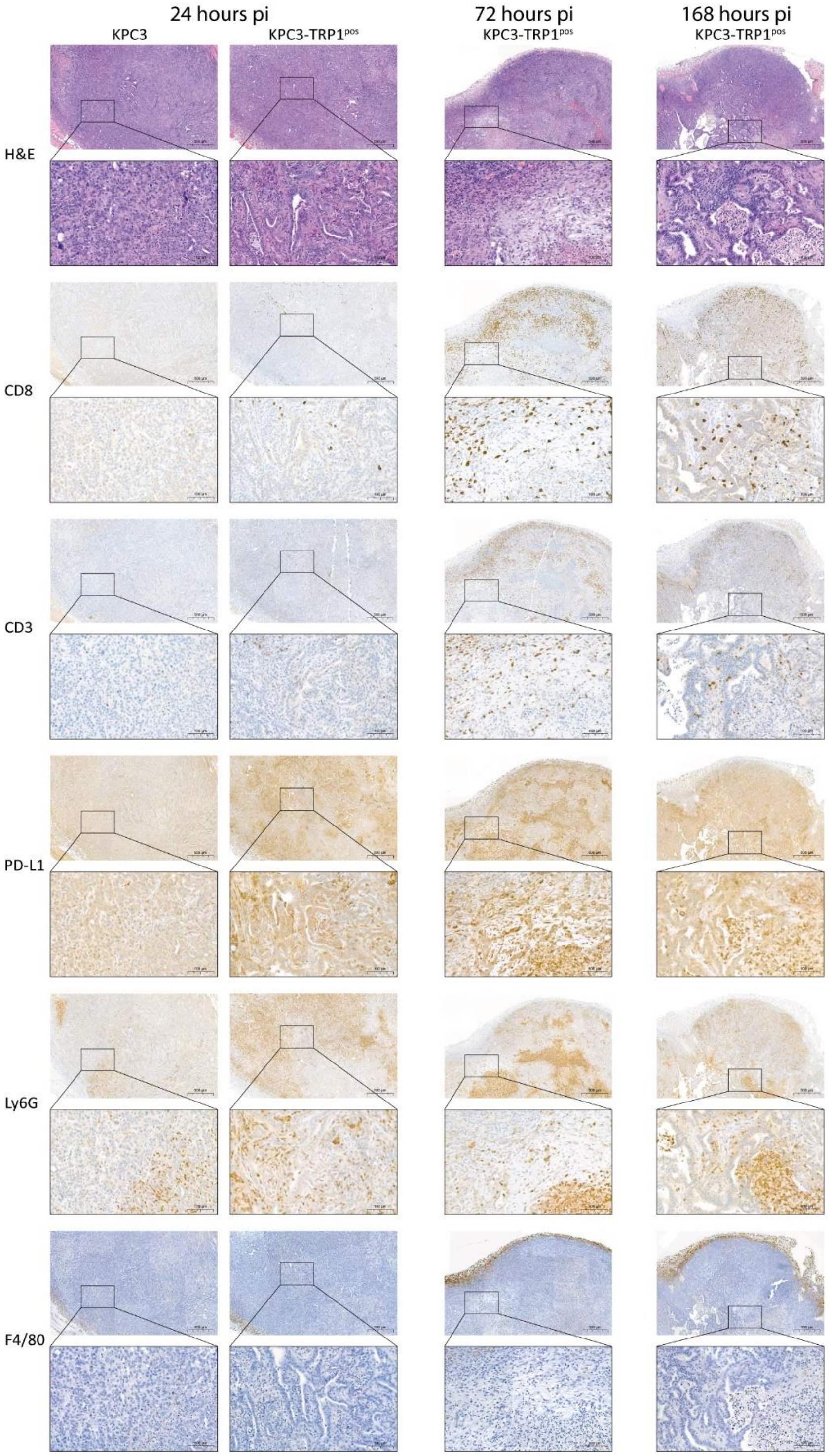

Figure S4. Therapy effects of CD3xTRP1 on KPC3-TRP1 and KPC3 tumors over time. Immunohistochemistry on 5 µm sections of FFPE KPC3-TRP1 and KPC3 tumors of contralaterally tumor-bearing C57BL/6J mice treated with 12.5 µg [^{111}In]In/[^{125}I]I-CD3xTRP1 at 24, 72, and 168 hours post intraperitoneal injection. Representative images of H&E, CD8, CD3, PD-L1, Ly6G, and F4/80 stains. The scale bars represents 500 µm, or 100 µm in insert.

31

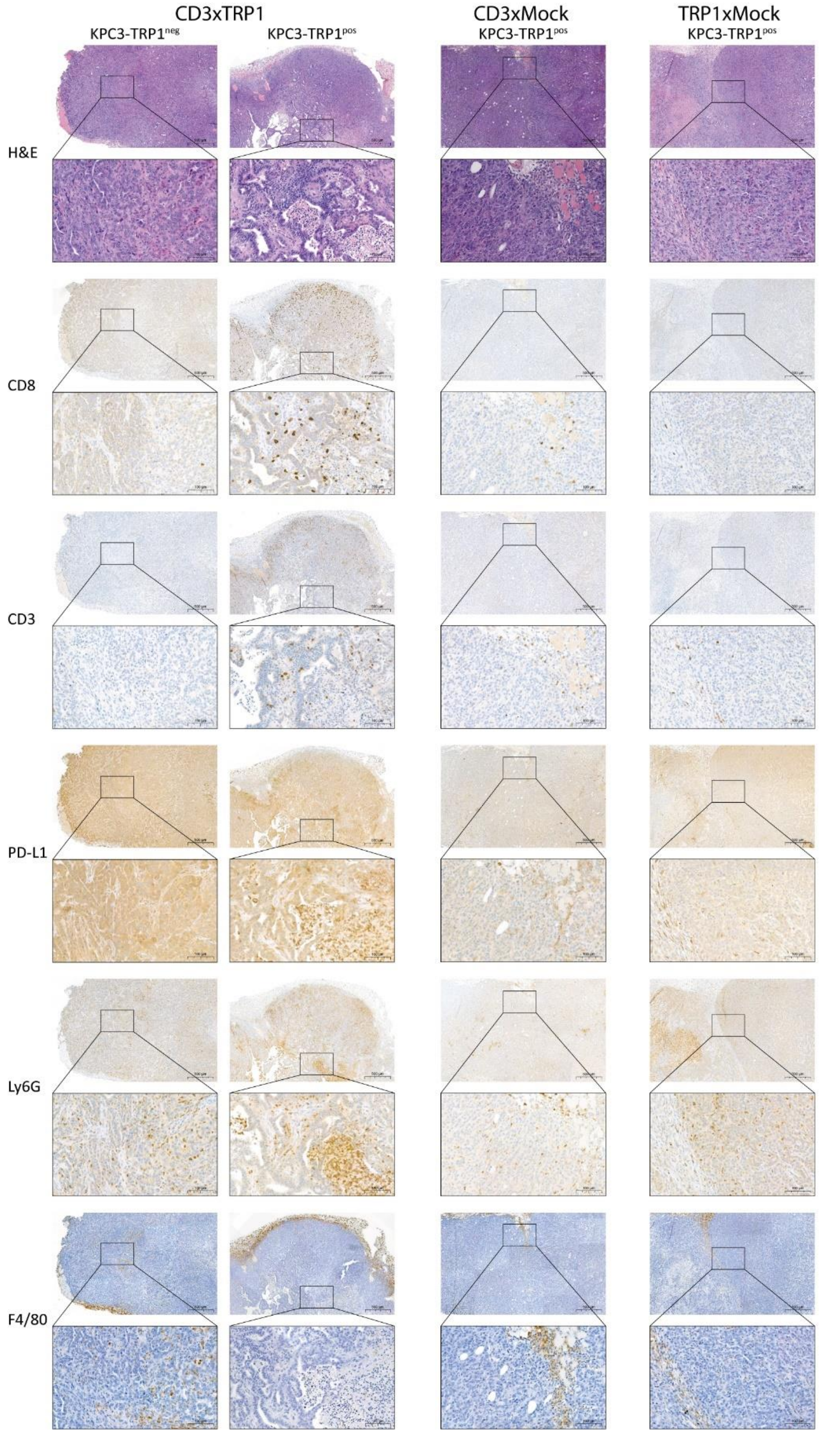

Figure S5. Therapy effects of CD3xTRP1, CD3xMock, and TRP1xMock on KPC3-TRP1 tumors. Immunohistochemistry on 5 µm sections of FFPE KPC3 and KPC3-TRP1 tumors of contralaterally tumor-bearing C57BL/6J mice treated with 12.5 µg [^{111}In]In/[^{125}I]I-CD3xTRP1, [^{111}In]In/[^{125}I]I-CD3xMock, or [^{111}In]In/[^{125}I]I-TRP1xMock at 168 hours post intraperitoneal injection. Representative images of H&E, CD8, CD3, PD-L1, Ly6G, and F4/80 stains. The scale bars represents 500 µm, or 100 µm in insert.

Table S1. *Ex vivo* biodistribution analysis of [^{111}In]In-CD3xTRP1, [^{111}In]In-CD3xMock, and [^{111}In]In-TRP1xMock in KPC3-TRP1 and KPC3 tumor-bearing C57BL/6J mice at 24, 72, and 168 hours pi, and [^{111}In]In-CD3xTRP1 in B16F10 tumor-bearing C57BL/6J mice. Mean results, standard deviation, and sample size per tissue are shown in %ID/g (mean ± sd (n=x)).

**24 hours pi**	**[^{111}In]In-CD3xTRP1**	**[^{111}In]In-CD3xMock**	**[^{111}In]In-TRP1xMock**
	(%ID/g)	(%ID/g)	(%ID/g)
**Blood**	22.3 ± 2.1 (n=5)	28.6 ± 7.8 (n=7)	32.1 ± 4.7 (n=6)
**Skin (pigmented)**	18.3 ± 0.0 (n=1)	11.5 ± 6.0 (n=5)	13.7 ± 1.3 (n=3)
**Skin (non-pigmented)**	9.6 ± 4.5 (n=5)	11.3 ± 4.8 (n=7)	15.7 ± 12.3 (n=6)
**Brown adipose tissue**	3.4 ± 0.5 (n=5)	4.3 ± 1.5 (n=7)	5.5 ± 1.6 (n=6)
**Lymph nodes**	50.3 ± 14.8 (n=5)	50.1 ± 19.2 (n=7)	19.2 ± 15.2 (n=6)
**KPC3**	8.3 ± 1.3 (n=4)	13.0 ± 8.4 (n=7)	10.6 ± 2.2 (n=5)
**KPC3-TRP1**	37.7 ± 5.3 (n=5)	10.0 ± 3.2 (n=6)	54.8 ± 15.3 (n=6)
**Muscle**	1.3 ± 0.2 (n=5)	1.5 ± 0.4 (n=7)	2.0 ± 0.5 (n=6)
**Thymus**	8.0 ± 1.0 (n=5)	10.8 ± 2.3 (n=7)	7.1 ± 2.1 (n=6)
**Heart**	5.3 ± 0.5 (n=5)	6.2 ± 2.2 (n=7)	7.8 ± 1.9 (n=6)
**Lung**	10.9 ± 1.9 (n=5)	13.4 ± 3.5 (n=7)	15.4 ± 5.0 (n=6)
**Spleen**	29.0 ± 3.9 (n=5)	48.7 ± 14.7 (n=7)	5.1 ± 1.1 (n=6)
**Pancreas**	4.8 ± 0.7 (n=5)	5.7 ± 1.5 (n=7)	6.8 ± 1.5 (n=6)
**Stomach**	3.2 ± 0.5 (n=5)	4.0 ± 1.1 (n=7)	4.9 ± 1.0 (n=6)
**Duodenum**	5.1 ± 0.8 (n=5)	7.0 ± 2.0 (n=7)	5.9 ± 1.3 (n=6)
**Colon**	2.7 ± 0.2 (n=5)	3.8 ± 1.3 (n=7)	4.0 ± 0.6 (n=6)
**Liver**	3.3 ± 0.5 (n=5)	4.3 ± 0.9 (n=7)	4.4 ± 0.6 (n=6)
**Kidney**	8.9 ± 0.8 (n=5)	10.4 ± 2.8 (n=7)	12.1 ± 2.0 (n=6)
**Prostate**	5.9 ± 1.6 (n=5)	8.5 ± 3.4 (n=7)	9.0 ± 2.8 (n=6)
**Bone marrow**	9.9 ± 2.6 (n=5)	12.8 ± 4.0 (n=7)	11.3 ± 3.3 (n=6)
**Bone**	2.2 ± 0.1 (n=5)	3.1 ± 0.8 (n=7)	2.8 ± 0.7 (n=6)

	KPC3-TRP1 and KPC3			B16F10
**72 hours pi**	**[^{111}In]In-CD3xTRP1**	**[^{111}In]In-CD3xMock**	**[^{111}In]In-TRP1xMock**	**[^{111}In]In-CD3xTRP1**
	(%ID/g)	(%ID/g)	(%ID/g)	(%ID/g)
**Blood**	16.3 ± 5.4 (n=5)	19.9 ± 4.9 (n=7)	23.8 ± 3.0 (n=6)	17.6 ± 2.3 (n=4)
**Skin (pigmented)**	9.0 ± 1.9 (n=2)	9.4 ± 2.5 (n=5)	12.9 ± 1.8 (n=2)	7.7 ± 2.2 (n=4)
**Skin (non-pigmented)**	8.3 ± 3.0 (n=5)	10.6 ± 4.8 (n=7)	15.5 ± 4.2 (n=6)	8.6 ± 5.1 (n=4)
**Brown adipose tissue**	2.7 ± 0.9 (n=5)	3.8 ± 1.0 (n=7)	4.0 ± 0.5 (n=6)	2.7 ± 0.5 (n=4)
**Lymph nodes**	45.3 ± 13.6 (n=5)	63.2 ± 17.2 (n=7)	11.5 ± 1.4 (n=6)	30.2 ± 11.3 (n=4)
**KPC3**	6.2 ± 2.1 (n=5)	9.1 ± 2.7 (n=7)	11.2 ± 1.4 (n=6)	n/a
**KPC3-TRP1 or B16F10**	33.5 ± 15.4 (n=5)	11.5 ± 7.3 (n=7)	74.2 ± 15.4 (n=6)	25.1 ± 15.1 (n=4)
**Muscle**	1.0 ± 0.3 (n=5)	1.2 ± 0.3 (n=7)	1.8 ± 0.3 (n=6)	0.7 ± 0.1 (n=4)
**Thymus**	10.0 ± 3.1 (n=5)	13.4 ± 3.4 (n=7)	5.2 ± 1.0 (n=6)	10.9 ± 2.1 (n=4)
**Heart**	3.6 ± 1.2 (n=5)	4.4 ± 1.1 (n=7)	5.0 ± 0.9 (n=6)	4.1 ± 0.9 (n=4)
**Lung**	8.7 ± 3.9 (n=5)	10.9 ± 2.7 (n=7)	11.4 ± 2.5 (n=6)	7.4 ± 1.3 (n=4)
**Spleen**	23.8 ± 10.4 (n=5)	46.4 ± 17.7 (n=7)	5.5 ± 0.5 (n=6)	30.9 ± 8.2 (n=4)
**Pancreas**	2.7 ± 0.9 (n=5)	3.3 ± 0.6 (n=7)	3.7 ± 0.5 (n=6)	2.6 ± 0.3 (n=4)
**Stomach**	2.3 ± 0.9 (n=5)	2.8 ± 0.6 (n=7)	3.0 ± 0.8 (n=6)	1.8 ± 0.5 (n=4)
**Duodenum**	4.4 ± 1.8 (n=5)	5.5 ± 1.4 (n=7)	3.3 ± 0.7 (n=6)	3.8 ± 0.5 (n=4)
**Colon**	1.7 ± 0.5 (n=5)	2.4 ± 0.6 (n=7)	2.2 ± 0.5 (n=6)	1.7 ± 0.3 (n=4)
**Liver**	3.8 ± 1.2 (n=5)	4.4 ± 1.0 (n=7)	3.9 ± 0.3 (n=6)	5.1 ± 0.7 (n=4)
**Kidney**	6.5 ± 2.0 (n=5)	8.2 ± 1.8 (n=7)	9.9 ± 1.1 (n=6)	6.6 ± 0.7 (n=4)
**Prostate**	3.5 ± 1.5 (n=5)	4.2 ± 1.4 (n=7)	5.4 ± 1.0 (n=6)	3.0 ± 0.6 (n=4)
**Bone marrow**	5.3 ± 1.7 (n=5)	8.7 ± 2.4 (n=7)	6.8 ± 1.0 (n=6)	4.8 ± 0.8 (n=4)
**Bone**	1.7 ± 0.6 (n=5)	2.6 ± 0.8 (n=7)	2.6 ± 0.5 (n=6)	1.5 ± 0.2 (n=4)

168 hours pi	[^{111}In]In-CD3xTRP1	[^{111}In]In-CD3xMock	[^{111}In]In-TRP1xMock
	(%ID/g)	(%ID/g)	(%ID/g)
**Blood**	7.8 ± 3.7 (n=6)	11.9 ± 3.5 (n=5)	9.4 ± 2.6 (n=6)
**Skin (pigmented)**	3.8 ± 2.1 (n=5)	4.0 ± 1.6 (n=3)	6.4 ± 1.2 (n=6)
**Skin (non-pigmented)**	4.9 ± 2.9 (n=6)	6.1 ± 3.3 (n=5)	8.5 ± 2.5 (n=7)
**Brown adipose tissue**	1.6 ± 0.7 (n=6)	2.8 ± 1.0 (n=5)	2.1 ± 0.6 (n=7)
**Lymph nodes**	26.3 ± 10.7 (n=6)	48.9 ± 30.3 (n=5)	9.7 ± 2.2 (n=7)
**KPC3**	3.3 ± 1.5 (n=6)	5.2 ± 1.9 (n=4)	4.2 ± 0.8 (n=7)
**KPC3-TRP1**	15.1 ± 5.8 (n=6)	5.2 ± 1.7 (n=5)	32.0 ± 10.1 (n=7)
**Muscle**	0.6 ± 0.3 (n=6)	0.7 ± 0.3 (n=5)	0.7 ± 0.1 (n=7)
**Thymus**	7.7 ± 3.0 (n=6)	11.5 ± 3.6 (n=5)	2.5 ± 0.4 (n=7)
**Heart**	2.0 ± 0.9 (n=6)	2.7 ± 0.9 (n=5)	2.3 ± 0.6 (n=7)
**Lung**	4.0 ± 2.0 (n=6)	5.9 ± 2.0 (n=5)	4.3 ± 0.6 (n=7)
**Spleen**	13.9 ± 7.0 (n=6)	27.6 ± 11.0 (n=5)	2.8 ± 0.5 (n=7)
**Pancreas**	1.7 ± 0.7 (n=6)	2.3 ± 0.9 (n=5)	1.7 ± 0.4 (n=7)
**Stomach**	1.1 ± 0.5 (n=6)	1.8 ± 0.7 (n=5)	1.3 ± 0.4 (n=7)
**Duodenum**	2.5 ± 1.1 (n=6)	4.6 ± 1.4 (n=5)	2.0 ± 0.6 (n=7)
**Colon**	0.9 ± 0.4 (n=6)	1.4 ± 0.5 (n=5)	1.1 ± 0.3 (n=7)
**Liver**	2.4 ± 1.0 (n=6)	3.4 ± 1.0 (n=5)	2.2 ± 0.3 (n=7)
**Kidney**	3.8 ± 1.5 (n=6)	5.2 ± 1.4 (n=5)	5.1 ± 0.9 (n=7)
**Prostate**	1.7 ± 0.7 (n=6)	2.6 ± 1.0 (n=5)	2.2 ± 0.4 (n=7)
**Bone marrow**	2.7 ± 1.1 (n=6)	5.0 ± 1.7 (n=5)	2.7 ± 0.6 (n=7)
**Bone**	0.9 ± 0.3 (n=6)	1.6 ± 0.5 (n=5)	1.1 ± 0.2 (n=7)

Table S2. Absolute accumulated CD3xTRP1, CD3xMock, and TRP1xMock as observed with *ex vivo* biodistribution of [^{111}In]In-CD3xTRP1 in KPC3-TRP1 and KPC3 tumor-bearing C57BL/6J mice at 24, 72, and 168 hours pi. The %ID which is not corrected for tissue weight is shown only for those tissues that were excised as a whole. Mean results, standard deviation, and sample size per tissue are shown in %ID (mean ± sd (n=x)).

	**[^{111}In]In-CD3xTRP1**			**[^{111}In]In-CD3xMock**			**[^{111}In]In-TRP1xMock**		
	24 hours pi	72 hours pi	168 hours pi	24 hours pi	72 hours pi	168 hours pi	24 hours pi	72 hours pi	168 hours pi
	(%ID)	(%ID)	(%ID)	(%ID)	(%ID)	(%ID)	(%ID)	(%ID)	(%ID)
**KPC3**	0.5 ± 0.4 (n=5)	1.6 ± 1.1 (n=5)	1.2 ± 1.0 (n=6)	0.8 ± 0.6 (n=6)	1.2 ± 0.7 (n=7)	2.1 ± 1.2 (n=4)	1.3 ± 0.6 (n=6)	1.8 ± 0.6 (n=6)	2.4 ± 1.3 (n=7)
**KPC3-TRP1**	5.9 ± 2.2 (n=5)	6.5 ± 1.9 (n=5)	6.0 ± 2.9 (n=6)	1.9 ± 1.0 (n=7)	2.5 ± 2.3 (n=7)	3.6 ± 1.6 (n=5)	7.3 ± 3.6 (n=6)	12.8 ± 6.7 (n=6)	24.5 ± 5.1 (n=7)
**Spleen**	2.7 ± 0.3 (n=5)	2.6 ± 0.8 (n=5)	2.1 ± 0.9 (n=6)	4.8 ± 1.2 (n=7)	4.4 ± 1.2 (n=7)	3.8 ± 1.1 (n=5)	0.4 ± 0.1 (n=6)	0.5 ± 0.0 (n=6)	0.4 ± 0.1 (n=7)
**Lymph nodes**	0.2 ± 0.0 (n=5)	0.3 ± 0.1 (n=5)	0.3 ± 0.1 (n=6)	0.2 ± 0.1 (n=7)	0.4 ± 0.2 (n=7)	0.3 ± 0.1 (n=5)	0.1 ± 0.0 (n=5)	0.1 ± 0.0 (n=6)	0.1 ± 0.0 (n=7)

Table S3. *Ex vivo* biodistribution analysis of ^{125}I-labeled CD3xTRP1, CD3xMock, and TRP1xMock in KPC3-TRP1 and KPC3 tumor-bearing C57BL/6J mice at 24, 72, and 168 hours pi. Mean results, standard deviation, and sample size per tissue are shown in %ID/g (mean ± sd (n=x)).

**24 hours pi**	**[^{125}I]I-CD3xTRP1**	**[^{125}I]I-CD3xMock**	**[^{125}I]I-TRP1xMock**
	(%ID/g)	(%ID/g)	(%ID/g)
**Blood**	18.5 ± 2.1 (n=5)	22.5 ± 6.3 (n=7)	21.6 ± 3.2 (n=6)
**Skin (pigmented)**	14.7 ± 0.0 (n=1)	8.3 ± 3.9 (n=5)	8.9 ± 0.6 (n=3)
**Skin (non-pigmented)**	7.6 ± 3.6 (n=5)	8.3 ± 3.4 (n=7)	9.4 ± 6.4 (n=6)
**Brown adipose tissue**	2.8 ± 0.4 (n=5)	3.3 ± 1.1 (n=7)	3.6 ± 0.9 (n=6)
**Lymph nodes**	12.8 ± 2.2 (n=5)	12.0 ± 3.2 (n=7)	7.5 ± 5.2 (n=6)
**KPC3**	5.9 ± 0.9 (n=4)	8.6 ± 6.0 (n=7)	5.9 ± 1.2 (n=5)
**KPC3-TRP1**	17.0 ± 2.7 (n=5)	6.6 ± 2.0 (n=6)	23.3 ± 6.2 (n=6)
**Muscle**	1.1 ± 0.2 (n=5)	1.2 ± 0.4 (n=7)	1.3 ± 0.3 (n=6)
**Thymus**	3.7 ± 0.8 (n=5)	4.8 ± 1.2 (n=7)	4.5 ± 1.4 (n=6)
**Heart**	4.3 ± 0.4 (n=5)	4.9 ± 1.8 (n=7)	5.2 ± 1.4 (n=6)
**Lung**	8.8 ± 1.7 (n=5)	10.0 ± 2.8 (n=7)	10.0 ± 3.3 (n=6)
**Spleen**	5.9 ± 0.6 (n=5)	7.8 ± 2.4 (n=7)	2.7 ± 0.6 (n=6)
**Pancreas**	3.2 ± 0.4 (n=5)	3.8 ± 1.0 (n=7)	4.0 ± 0.9 (n=6)
**Stomach**	2.7 ± 1.0 (n=5)	2.9 ± 1.0 (n=7)	3.5 ± 0.6 (n=6)
**Duodenum**	4.7 ± 2.9 (n=5)	5.7 ± 3.9 (n=7)	3.8 ± 0.9 (n=6)
**Colon**	1.9 ± 0.2 (n=5)	2.6 ± 1.0 (n=7)	2.4 ± 0.4 (n=6)
**Liver**	2.1 ± 0.3 (n=5)	2.6 ± 0.6 (n=7)	2.6 ± 0.4 (n=6)
**Kidney**	5.3 ± 0.4 (n=5)	6.3 ± 1.8 (n=7)	6.1 ± 1.0 (n=6)
**Prostate**	5.1 ± 1.7 (n=5)	6.5 ± 2.5 (n=7)	5.9 ± 1.9 (n=6)
**Bone marrow**	5.8 ± 1.2 (n=5)	6.4 ± 2.0 (n=7)	6.5 ± 2.2 (n=6)
**Bone**	1.4 ± 0.1 (n=5)	1.8 ± 0.5 (n=7)	1.6 ± 0.4 (n=6)

**72 hours pi**	**[^{125}I]I-CD3xTRP1**	**[^{125}I]I-CD3xMock**	**[^{125}I]I-TRP1xMock**
	(%ID/g)	(%ID/g)	(%ID/g)
**Blood**	13.7 ± 4.5 (n=5)	16.1 ± 3.9 (n=7)	15.9 ± 2.1 (n=6)
**Skin (pigmented)**	6.1 ± 1.2 (n=2)	6.9 ± 1.6 (n=5)	7.5 ± 0.8 (n=2)
**Skin (non-pigmented)**	5.9 ± 2.4 (n=5)	8.0 ± 3.5 (n=7)	9.2 ± 2.5 (n=6)
**Brown adipose tissue**	2.2 ± 0.8 (n=5)	2.8 ± 0.7 (n=7)	2.5 ± 0.3 (n=6)
**Lymph nodes**	6.8 ± 2.0 (n=5)	8.1 ± 2.3 (n=7)	3.3 ± 0.3 (n=6)
**KPC3**	3.8 ± 1.4 (n=5)	5.5 ± 1.6 (n=7)	5.6 ± 0.9 (n=6)
**KPC3-TRP1**	7.6 ± 4.1 (n=5)	5.4 ± 1.6 (n=7)	19.9 ± 3.6 (n=6)
**Muscle**	0.9 ± 0.3 (n=5)	0.9 ± 0.2 (n=7)	1.2 ± 0.2 (n=6)
**Thymus**	2.5 ± 0.8 (n=5)	3.1 ± 0.7 (n=7)	2.8 ± 0.6 (n=6)
**Heart**	2.9 ± 1.0 (n=5)	3.4 ± 0.8 (n=7)	3.3 ± 0.6 (n=6)
**Lung**	6.7 ± 3.0 (n=5)	8.0 ± 2.0 (n=7)	7.3 ± 1.7 (n=6)
**Spleen**	3.2 ± 1.3 (n=5)	4.3 ± 1.2 (n=7)	2.1 ± 0.2 (n=6)
**Pancreas**	1.7 ± 0.4 (n=5)	2.0 ± 0.3 (n=7)	1.9 ± 0.3 (n=6)
**Stomach**	1.9 ± 0.7 (n=5)	2.2 ± 0.4 (n=7)	2.2 ± 0.5 (n=6)
**Duodenum**	2.1 ± 0.7 (n=5)	2.3 ± 0.5 (n=7)	1.9 ± 0.4 (n=6)
**Colon**	1.2 ± 0.4 (n=5)	1.4 ± 0.2 (n=7)	1.3 ± 0.3 (n=6)
**Liver**	1.9 ± 0.6 (n=5)	2.4 ± 0.8 (n=7)	2.0 ± 0.1 (n=6)
**Kidney**	3.9 ± 1.1 (n=5)	4.5 ± 1.4 (n=7)	4.7 ± 0.6 (n=6)
**Prostate**	2.5 ± 1.0 (n=5)	3.1 ± 0.8 (n=7)	3.2 ± 0.6 (n=6)
**Bone marrow**	2.6 ± 0.9 (n=5)	3.8 ± 0.9 (n=7)	3.4 ± 0.5 (n=6)
**Bone**	1.0 ± 0.3 (n=5)	1.3 ± 0.4 (n=7)	1.3 ± 0.2 (n=6)

**168 hours pi**	**[^{125}I]I-CD3xTRP1**	**[^{125}I]I-CD3xMock**	**[^{125}I]I-TRP1xMock**
	(%ID/g)	(%ID/g)	(%ID/g)
**Blood**	6.5 ± 3.1 (n=6)	9.3 ± 2.6 (n=5)	6.0 ± 1.7 (n=6)
**Skin (pigmented)**	2.5 ± 1.3 (n=5)	2.7 ± 1.1 (n=3)	3.3 ± 0.6 (n=6)
**Skin (non-pigmented)**	3.1 ± 1.7 (n=6)	4.1 ± 2.3 (n=5)	4.3 ± 1.3 (n=7)
**Brown adipose tissue**	1.2 ± 0.5 (n=6)	1.9 ± 0.8 (n=5)	1.2 ± 0.4 (n=7)
**Lymph nodes**	2.5 ± 1.1 (n=6)	4.7 ± 2.8 (n=5)	1.3 ± 0.2 (n=7)
**KPC3**	1.9 ± 0.9 (n=6)	2.6 ± 0.9 (n=4)	1.7 ± 0.3 (n=7)
**KPC3-TRP1**	3.1 ± 1.1 (n=6)	2.8 ± 0.9 (n=5)	6.2 ± 1.9 (n=7)
**Muscle**	0.4 ± 0.2 (n=6)	0.5 ± 0.2 (n=5)	0.4 ± 0.1 (n=7)
**Thymus**	1.4 ± 0.7 (n=6)	1.7 ± 0.3 (n=5)	0.9 ± 0.2 (n=7)
**Heart**	1.6 ± 0.8 (n=6)	2.0 ± 0.6 (n=5)	1.4 ± 0.4 (n=7)
**Lung**	2.9 ± 1.6 (n=6)	3.8 ± 1.3 (n=5)	2.3 ± 0.3 (n=7)
**Spleen**	1.3 ± 0.7 (n=6)	1.9 ± 0.8 (n=5)	0.6 ± 0.1 (n=7)
**Pancreas**	0.8 ± 0.4 (n=6)	1.2 ± 0.4 (n=5)	0.8 ± 0.2 (n=7)
**Stomach**	0.9 ± 0.3 (n=6)	1.6 ± 0.4 (n=5)	1.1 ± 0.2 (n=7)
**Duodenum**	1.0 ± 0.4 (n=6)	1.4 ± 0.4 (n=5)	1.0 ± 0.3 (n=7)
**Colon**	0.5 ± 0.3 (n=6)	0.8 ± 0.3 (n=5)	0.5 ± 0.2 (n=7)
**Liver**	1.0 ± 0.5 (n=6)	1.1 ± 0.4 (n=5)	0.8 ± 0.2 (n=7)
**Kidney**	2.2 ± 0.8 (n=6)	2.9 ± 0.8 (n=5)	2.0 ± 0.5 (n=7)
**Prostate**	1.3 ± 0.6 (n=6)	1.7 ± 0.7 (n=5)	1.2 ± 0.1 (n=7)
**Bone marrow**	0.9 ± 0.4 (n=6)	1.4 ± 0.6 (n=5)	0.8 ± 0.1 (n=7)
**Bone**	0.4 ± 0.1 (n=6)	0.6 ± 0.2 (n=5)	0.4 ± 0.1 (n=7)

Table S4. *In vivo* internalization shown as a ratio between ^{125}I-labeled and ^{111}In-labeled CD3xTRP1, CD3xMock, and TRP1xMock in KPC3-TRP1 and KPC3 tumor-bearing C57BL/6J mice at 24, 72, and 168 hours pi. Mean ^{125}I/^{111}In-ratio, standard deviation, and sample size per tissue are shown (mean ± sd (n=x)).

**24 hours pi**	**CD3xTRP1**	**CD3xMock**	**TRP1xMock**
	^{125}I/^{111}In-ratio	^{125}I/^{111}In-ratio	^{125}I/^{111}In-ratio
**Blood**	0.82 ± 0.02 (n=5)	0.79 ± 0.01 (n=7)	0.67 ± 0.02 (n=6)
**Skin (pigmented)**	0.80 ± 0.00 (n=1)	0.74 ± 0.04 (n=5)	0.66 ± 0.02 (n=3)
**Skin (non-pigmented)**	0.80 ± 0.01 (n=5)	0.74 ± 0.02 (n=7)	0.63 ± 0.04 (n=6)
**Brown adipose tissue**	0.84 ± 0.02 (n=5)	0.79 ± 0.03 (n=7)	0.66 ± 0.02 (n=6)
**Lymph nodes**	0.27 ± 0.04 (n=5)	0.25 ± 0.03 (n=7)	0.41 ± 0.05 (n=6)
**KPC3**	0.71 ± 0.01 (n=4)	0.66 ± 0.02 (n=7)	0.56 ± 0.01 (n=5)
**KPC3-TRP1**	0.45 ± 0.02 (n=5)	0.66 ± 0.01 (n=6)	0.43 ± 0.02 (n=6)
**Muscle**	0.82 ± 0.00 (n=5)	0.80 ± 0.02 (n=7)	0.64 ± 0.03 (n=6)
**Thymus**	0.45 ± 0.05 (n=5)	0.44 ± 0.05 (n=7)	0.64 ± 0.01 (n=6)
**Heart**	0.82 ± 0.02 (n=5)	0.79 ± 0.01 (n=7)	0.66 ± 0.02 (n=6)
**Lung**	0.80 ± 0.02 (n=5)	0.75 ± 0.02 (n=7)	0.65 ± 0.02 (n=6)
**Spleen**	0.20 ± 0.01 (n=5)	0.16 ± 0.01 (n=7)	0.52 ± 0.02 (n=6)
**Pancreas**	0.66 ± 0.02 (n=5)	0.65 ± 0.03 (n=7)	0.59 ± 0.02 (n=6)
**Stomach**	0.84 ± 0.30 (n=5)	0.77 ± 0.27 (n=7)	0.73 ± 0.04 (n=6)
**Duodenum**	0.98 ± 0.75 (n=5)	0.75 ± 0.37 (n=7)	0.64 ± 0.02 (n=6)
**Colon**	0.71 ± 0.04 (n=5)	0.68 ± 0.03 (n=7)	0.61 ± 0.03 (n=6)
**Liver**	0.63 ± 0.02 (n=5)	0.60 ± 0.04 (n=7)	0.59 ± 0.02 (n=6)
**Kidney**	0.60 ± 0.02 (n=5)	0.61 ± 0.02 (n=7)	0.50 ± 0.01 (n=6)
**Prostate**	0.85 ± 0.06 (n=5)	0.78 ± 0.03 (n=7)	0.65 ± 0.02 (n=6)
**Bone marrow**	0.59 ± 0.04 (n=5)	0.50 ± 0.03 (n=7)	0.57 ± 0.04 (n=6)
**Bone**	0.64 ± 0.02 (n=5)	0.58 ± 0.02 (n=7)	0.59 ± 0.03 (n=6)

72 hours pi	CD3xTRP1	CD3xMock	TRP1xMock
	$^{125}I/^{111}In$-ratio	$^{125}I/^{111}In$-ratio	$^{125}I/^{111}In$-ratio
**Blood**	0.84 ± 0.02 (n=5)	0.81 ± 0.03 (n=7)	0.67 ± 0.01 (n=6)
**Skin (pigmented)**	0.68 ± 0.01 (n=2)	0.74 ± 0.06 (n=5)	0.59 ± 0.02 (n=2)
**Skin (non-pigmented)**	0.70 ± 0.10 (n=5)	0.76 ± 0.06 (n=7)	0.60 ± 0.03 (n=6)
**Brown adipose tissue**	0.81 ± 0.03 (n=5)	0.76 ± 0.07 (n=7)	0.64 ± 0.01 (n=6)
**Lymph nodes**	0.15 ± 0.00 (n=5)	0.13 ± 0.02 (n=7)	0.29 ± 0.05 (n=6)
**KPC3**	0.62 ± 0.04 (n=5)	0.60 ± 0.08 (n=7)	0.50 ± 0.03 (n=6)
**KPC3-TRP1**	0.22 ± 0.03 (n=5)	0.53 ± 0.11 (n=7)	0.27 ± 0.04 (n=6)
**Muscle**	0.85 ± 0.05 (n=5)	0.80 ± 0.07 (n=7)	0.65 ± 0.02 (n=6)
**Thymus**	0.25 ± 0.04 (n=5)	0.24 ± 0.06 (n=7)	0.53 ± 0.04 (n=6)
**Heart**	0.81 ± 0.01 (n=5)	0.80 ± 0.06 (n=7)	0.65 ± 0.02 (n=6)
**Lung**	0.76 ± 0.03 (n=5)	0.74 ± 0.08 (n=7)	0.63 ± 0.01 (n=6)
**Spleen**	0.14 ± 0.02 (n=5)	0.10 ± 0.03 (n=7)	0.37 ± 0.02 (n=6)
**Pancreas**	0.68 ± 0.17 (n=5)	0.60 ± 0.03 (n=7)	0.51 ± 0.03 (n=6)
**Stomach**	0.85 ± 0.14 (n=5)	0.78 ± 0.08 (n=7)	0.72 ± 0.03 (n=6)
**Duodenum**	0.48 ± 0.04 (n=5)	0.43 ± 0.06 (n=7)	0.57 ± 0.04 (n=6)
**Colon**	0.69 ± 0.03 (n=5)	0.61 ± 0.06 (n=7)	0.59 ± 0.02 (n=6)
**Liver**	0.52 ± 0.04 (n=5)	0.59 ± 0.33 (n=7)	0.51 ± 0.02 (n=6)
**Kidney**	0.61 ± 0.05 (n=5)	0.54 ± 0.09 (n=7)	0.48 ± 0.02 (n=6)
**Prostate**	0.78 ± 0.13 (n=5)	0.76 ± 0.07 (n=7)	0.60 ± 0.04 (n=6)
**Bone marrow**	0.48 ± 0.04 (n=5)	0.45 ± 0.08 (n=7)	0.50 ± 0.05 (n=6)
**Bone**	0.58 ± 0.03 (n=5)	0.52 ± 0.05 (n=7)	0.49 ± 0.03 (n=6)

168 hours pi	CD3xTRP1	CD3xMock	TRP1xMock
	$^{125}I/^{111}In$-ratio	$^{125}I/^{111}In$-ratio	$^{125}I/^{111}In$-ratio
**Blood**	0.84 ± 0.03 (n=6)	0.79 ± 0.03 (n=5)	0.64 ± 0.01 (n=6)
**Skin (pigmented)**	0.67 ± 0.05 (n=5)	0.68 ± 0.04 (n=3)	0.51 ± 0.04 (n=6)
**Skin (non-pigmented)**	0.63 ± 0.09 (n=6)	0.65 ± 0.07 (n=5)	0.50 ± 0.04 (n=7)
**Brown adipose tissue**	0.76 ± 0.12 (n=6)	0.66 ± 0.06 (n=5)	0.55 ± 0.03 (n=7)
**Lymph nodes**	0.09 ± 0.02 (n=6)	0.10 ± 0.02 (n=5)	0.14 ± 0.02 (n=7)
**KPC3**	0.57 ± 0.04 (n=6)	0.51 ± 0.03 (n=4)	0.39 ± 0.03 (n=7)
**KPC3-TRP1**	0.21 ± 0.03 (n=6)	0.55 ± 0.06 (n=5)	0.20 ± 0.02 (n=7)
**Muscle**	0.74 ± 0.04 (n=6)	0.79 ± 0.09 (n=5)	0.57 ± 0.01 (n=7)
**Thymus**	0.19 ± 0.06 (n=6)	0.16 ± 0.03 (n=5)	0.37 ± 0.05 (n=7)
**Heart**	0.80 ± 0.03 (n=6)	0.74 ± 0.04 (n=5)	0.60 ± 0.02 (n=7)
**Lung**	0.73 ± 0.05 (n=6)	0.65 ± 0.04 (n=5)	0.54 ± 0.01 (n=7)
**Spleen**	0.10 ± 0.01 (n=6)	0.07 ± 0.00 (n=5)	0.22 ± 0.02 (n=7)
**Pancreas**	0.50 ± 0.07 (n=6)	0.54 ± 0.09 (n=5)	0.47 ± 0.02 (n=7)
**Stomach**	0.90 ± 0.10 (n=6)	1.10 ± 0.53 (n=5)	0.81 ± 0.14 (n=7)
**Duodenum**	0.38 ± 0.03 (n=6)	0.31 ± 0.02 (n=5)	0.48 ± 0.03 (n=7)
**Colon**	0.59 ± 0.07 (n=6)	0.54 ± 0.05 (n=5)	0.50 ± 0.04 (n=7)
**Liver**	0.39 ± 0.07 (n=6)	0.33 ± 0.02 (n=5)	0.37 ± 0.03 (n=7)
**Kidney**	0.62 ± 0.18 (n=6)	0.56 ± 0.02 (n=5)	0.39 ± 0.03 (n=7)
**Prostate**	0.77 ± 0.14 (n=6)	0.65 ± 0.06 (n=5)	0.55 ± 0.06 (n=7)
**Bone marrow**	0.35 ± 0.07 (n=6)	0.26 ± 0.06 (n=5)	0.28 ± 0.04 (n=7)
**Bone**	0.43 ± 0.03 (n=6)	0.36 ± 0.03 (n=5)	0.34 ± 0.02 (n=7)

Table S5. Serum fraction of blood circulating [^{111}In]In-CD3xTRP1 and control bsAbs over time. Serum fraction of blood circulating [^{111}In]In-CD3xTRP1, [^{111}In]In-CD3xMock, and [^{111}In]In-TRP1xMock in C57BL/6J mice bearing KPC3-TRP1 and KPC3 tumors that received 12.5 µg [^{111}In]In-bsAb intraperitoneally at 24, 72, and 168 hours pi. Mean ^{111}In serum fraction, standard deviation, and sample size are shown (mean ± sd (n=x)).

	**24 hours pi**	**72 hours pi**	**168 hours pi**
	(%)	(%)	(%)
**CD3xTRP1**	75.4 ± 5.4 (n=5)	73.4 ± 3.7 (n=5)	80.7 ± 5.9 (n=6)
**CD3xMock**	76.8 ± 5.1 (n=6)	80.6 ± 6.9 (n=7)	81.0 ± 7.4 (n=5)
**TRP1xMock**	79.5 ± 4.3 (n=6)	77.6 ± 3.5 (n=6)	78.3 ± 6.0 (n=7)
