# Supplementary material for: Longitudinal evaluation of the biodistribution and cellular internalization of the bispecific CD3xTRP1 antibody in syngeneic mouse tumor models
Source: J Immunother Cancer. 2023 Oct 29;11(10):e007596. doi: 10.1136/jitc-2023-007596 (PMC10619024; doi:10.1136/jitc-2023-007596)
Supplement: Supplementary data [file jitc-2023-007596supp002.pdf]

Longitudinal evaluation of the biodistribution and cellular internalization of the bispecific CD3xTRP1 antibody in syngeneic mouse tumor models

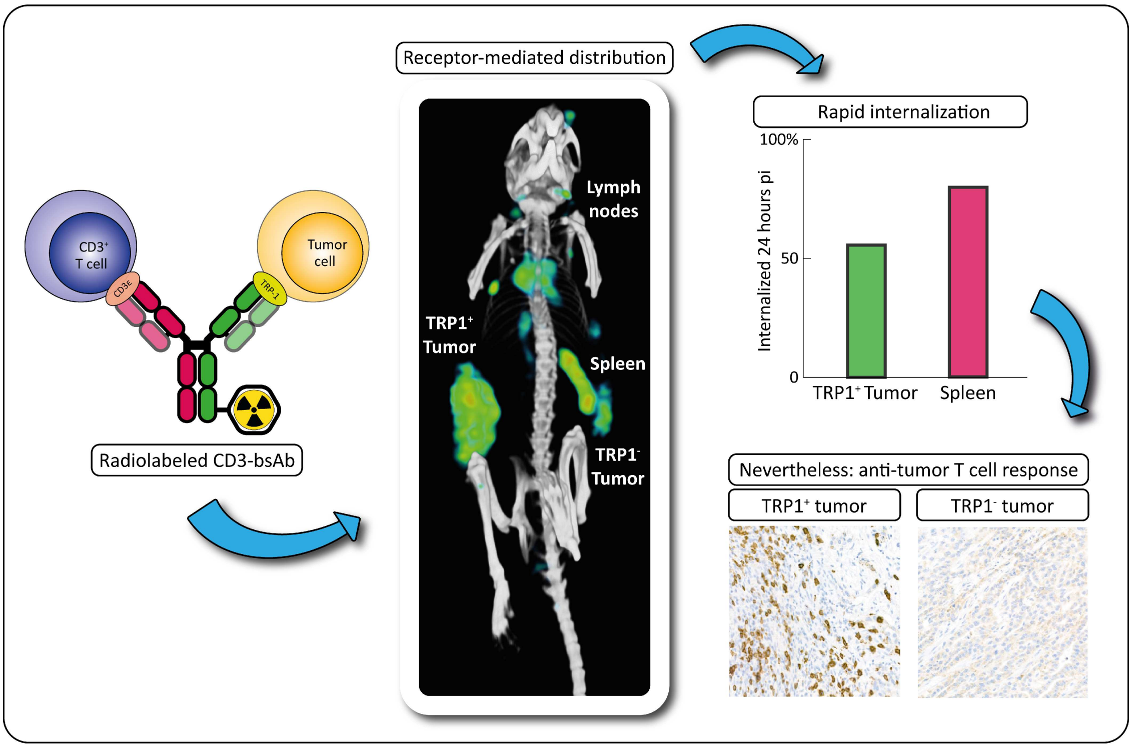

Authors
Gerwin G.W. Sandker
Jim Middelburg
Evienne Wilbrink
Janneke Molkenboer-Kuenen
Erik H.J.G. Aarntzen
Thorbald van Hall
Sandra Heskamp

Correspondence
Sandra.Heskamp@radboudumc.nl

In brief
CD3xTRP1 bispecific antibody distributes specifically to TRP1⁺ tumors and CD3-rich tissues through receptor-mediated uptake and not through trafficking by CD3⁺ T cells. Although CD3xTRP1 rapidly internalized in TRP1⁺ tumors and CD3-rich tissues it still induced effective anti-tumor immune responses.
